# Reevaluation of the Acute Cystitis Symptom Score, a Self-Reporting Questionnaire. Part II. Patient-Reported Outcome Assessment

**DOI:** 10.3390/antibiotics7020043

**Published:** 2018-05-21

**Authors:** Jakhongir F. Alidjanov, Kurt G. Naber, Ulugbek A. Abdufattaev, Adrian Pilatz, Florian M. Wagenlehner

**Affiliations:** 1 State Institution “Republican Specialized Scientific-Practical Medical Center of Urology”, Tashkent 100109, Uzbekistan; jakhonghir@hotmail.com (J.F.A.); abdufattaev@gmail.com (U.A.A.); 2 Clinic of Urology, Pediatric Urology, and Andrology, Justus Liebig University, 35392 Giessen, Germany; pilatz@t-online.de (A.P.); Florian.Wagenlehner@chiru.med.uni-giessen.de (F.M.W.); 3 Department of Urology, School of Medicine, Technical University of Munich, 80333 Munich, Germany

**Keywords:** cystitis, female, quality of life, urinary tract infection, Acute Cystitis Symptom Score, questionnaire, patient-reported outcome

## Abstract

This study aimed to reevaluate the Acute Cystitis Symptom Score (ACSS). The ACSS is a self-reporting questionnaire for the clinical diagnosis of acute uncomplicated cystitis (AC) and the assessment of symptomatic changes after therapy in female patients with AC. The part II of the present study was to reevaluate the utility of the different domains of the ACSS after therapy. The applicability of these domains in assessing changes in symptoms, as a function of time, in this population was investigated. The ACSS was evaluated in 48 female patients (mean age 31.1 ± 10.6) in the Uzbek and Russian languages, who returned after therapy and filled in part B of the ACSS, which corresponds to part A with the additional “Dynamics” domain. Descriptive statistics were used, where suitable. The reduction of typical symptoms and quality of life assessment between first and follow-up visit correlated significantly with answers in the “Dynamics” domain. *Success/Cure* and *Non-success/Failure* could be clearly differentiated by the scores obtained in “Typical” and “Quality of Life” domains. The ACSS has proven to be a useful instrument to clinically diagnose AC in women. It is also a suitable instrument for patient-reported outcome measures, with applicability both in daily practice and clinical studies. Slight modifications in the “Dynamics” domain will even increase the applicability.

## 1. Introduction

The Acute Cystitis Symptom Score (ACSS) was developed and validated as a simple and self-reporting questionnaire for diagnosing acute uncomplicated cystitis (AC) in female patients by assessing typical and differential symptoms, quality of life, and additional health conditions, which may play an important role in such a clinical setting. The evaluation in 286 women in Uzbek and Russian languages, which also included the results of the 58 women in whom the preliminary slightly different questionnaire: Urinary Symptoms and the Quality of Life Assessment Tool (USQOLAT), was applied, has been published earlier [1,2]. Part I of the report on the evaluation of the 228 women in whom only the current ACSS in Uzbek and Russian language was applied, deals mainly with the diagnostics of AC [3]. The studies were performed in Uzbekistan. Although Uzbek is the official language, Russian as the second language remains in widespread use (https://en.wikipedia.org/wiki/Uzbekistan), with the majority of the population speaking both languages. Both evaluations revealed significant differences in the scores in the domain with typical symptoms and with the quality of life between female patients with AC and controls. As an optimal threshold to predict AC, a total score of six points in the domain of typical symptoms can be established. In part of the patients, a follow-up visit was performed. The overall symptom score decreased significantly when comparing before and after therapy [1,2].

The present study refers to the subgroup of 48 female patients with AC, in whom part A (first visit) and part B (follow-up visit) of the current ACSS in Uzbek and Russian languages were applied, because they also participated in a follow up visit, to study in more detail the suitability of the ACSS as a practical instrument for patient-reported outcome assessment.

## 2. Patients and Methods

### 2.1. Clinical Procedures

The development of the ACSS and the total study population were described earlier in details [3]. A subgroup of 48 patients (mean age 31.10, standard deviation 10.64, range 19–63) who returned to a follow-up visit was also asked to fill in the part B (follow-up form) of the ACSS (Supplementary Figures S1 and S2). All participants signed written informed consent before filling in the questionnaire. Data from filled-in paper-form questionnaires were then recorded in an electronic form using PC software specially developed for the purpose of recording, storing, and processing inputted data (e-USQOLAT). Besides medical history and clinical evaluation in all patients, microscopic analysis of the urinary sediment and urine culture were performed at the first visit. Otherwise, the study was performed under conditions of clinical practice, where no specific treatment modalities and follow up visits were required to be included in the study.

At the follow-up visit, the patients were asked to fill in the follow-up part (part B) of the ACSS containing the same questions as the diagnostic part (part A) in the domains of typical and differential symptoms, quality of life, and additional health conditions. In addition, part B includes a domain “Dynamics” with five questions concerning overall evolution and changes of the symptomatology (Table 1). During the follow-up visit in 42/48 (87.5%) patients in addition to clinical evaluation also microscopic urinalysis was performed, whereas urine culture was performed only to the physician’s discretion, e.g., in case of treatment failure.

### 2.2. Statistical Analysis

Ordinary descriptive statistics were used for demographic characteristics of the study respondents. Calculation of Cronbach’s alpha [4] was used for assessment of internal consistency for the part B of the ACSS. Nonparametric Wilcoxon’s signed rank test [5] was used for comparative analysis of variables for related samples and parametric paired *t*-test was used for a reassessment of statistical significance [6]. Means and 95% confidence intervals were calculated. A *p*-value equal or lower than 0.05 was considered statistically significant. Substantive significance (effect size) was estimated by the modified correlation coefficient (*r*) proposed by Rosenthal and Rosnow [7] using Z value retrieved from the Wilcoxon’s signed-rank test. IBM SPSS for Windows, Version 22.0 (IBM Corp., Armonk, NY, USA) was used for statistical analysis and graphical presentations of the results.

## 3. Results

### 3.1. Study Population

Of a total of 107 patients with AC, 48 (44.9%) had a subsequent follow-up visit after 5.08 ± 2.71 (range 3–18; median 4.00; IQR (IQR—interquartile range) (3.25–6.00) days of therapy. The Uzbek Cyrillic version of the ACSS was filled in by 38/48 (79.2%) of the patients. The remaining 10/48 patients (20.8%) filled in the Russian version. The mean (SD) age of the patients was 31.1 (10.6) years; range 19 to 63 years; nine (18.8%) of them were pregnant. During the follow-up visit, 42 of the (87.5%) 48 subjects had a microscopic urinalysis performed. However, urine culture was done at the physician’s discretion.

### 3.2. Dynamics

The responses of the 48 female patients treated for AC in the “Dynamics” domain at the follow-up visit (ACSS part B) are shown in Table 1. All symptoms went off in 12 (25.0%); the majority of symptoms went off in 26 (52.2%); some symptoms still remained in 8 (16.7%), and all symptoms remained in two (4.2%) patients. In none of the patients, the condition declined.

### 3.3. Reliability of the Follow-Up form of the ACSS

Internal consistency of the follow-up form of the ACSS, including the “Dynamics” domain, was 0.92 (95% CI: 0.89 to 0.95). Values of internal consistency for ACSS “if item deleted” and item-total correlations are presented in Table 2.

### 3.4. ACSS Scores at First and Follow-Up Visits

**Figure 1:**
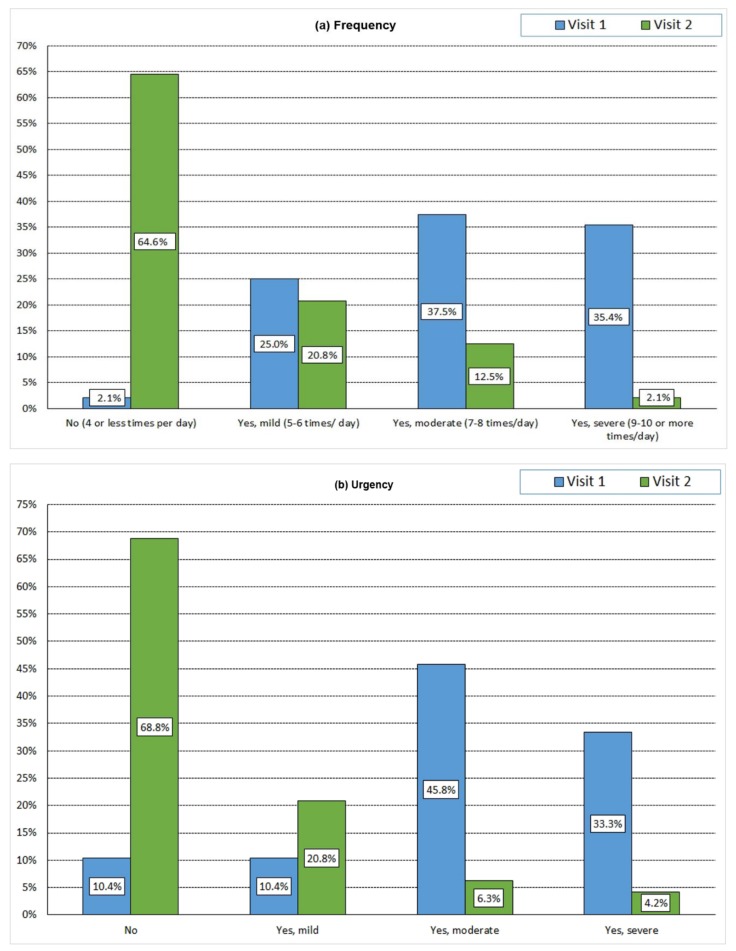

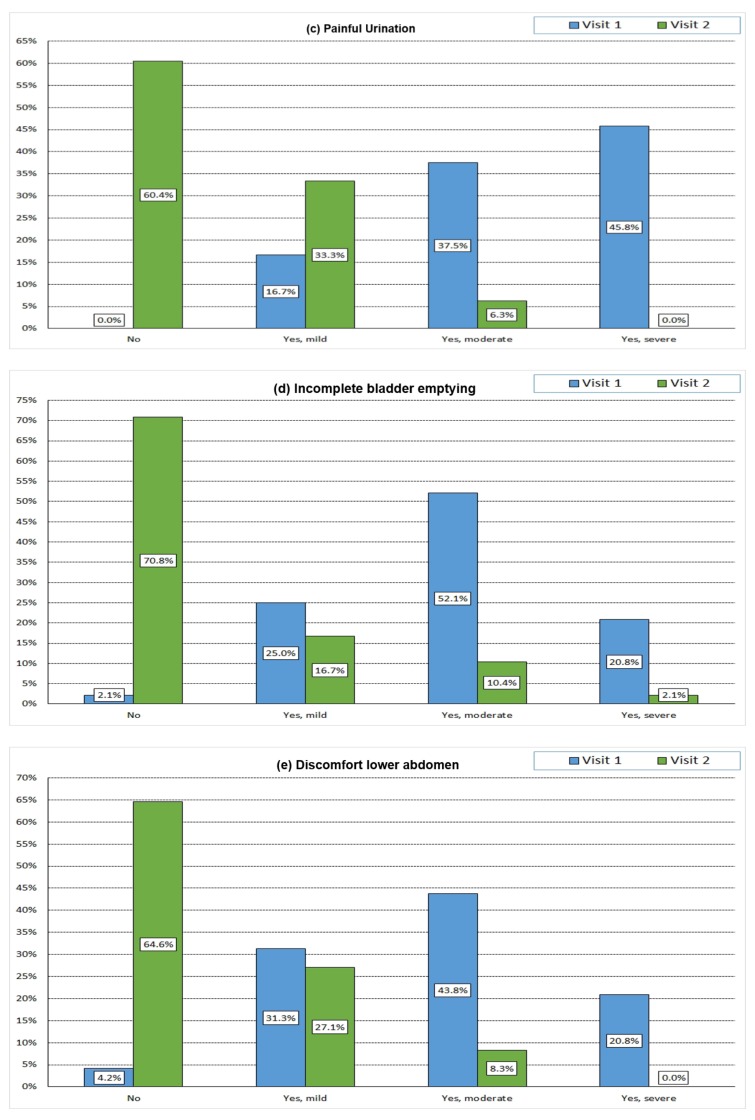

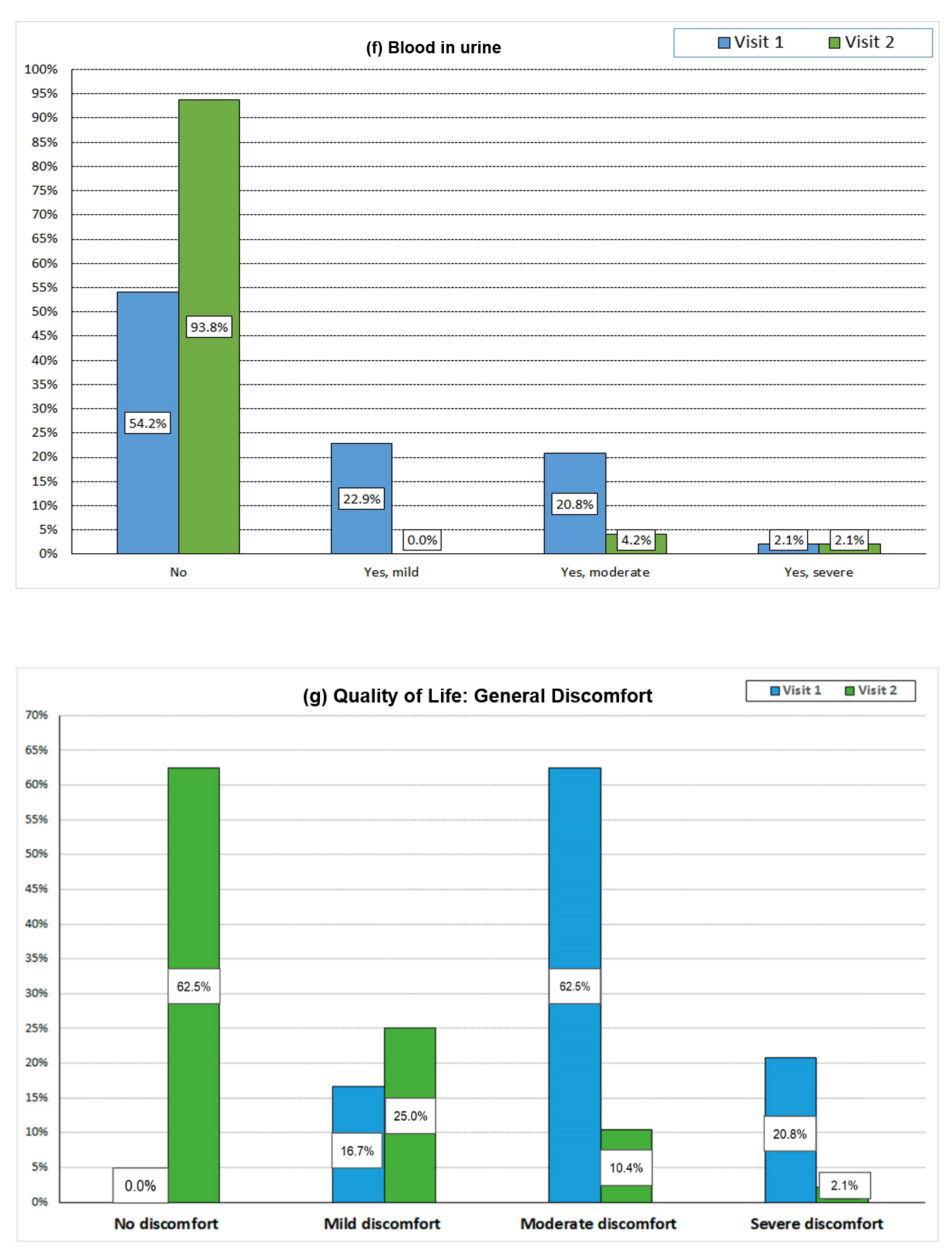

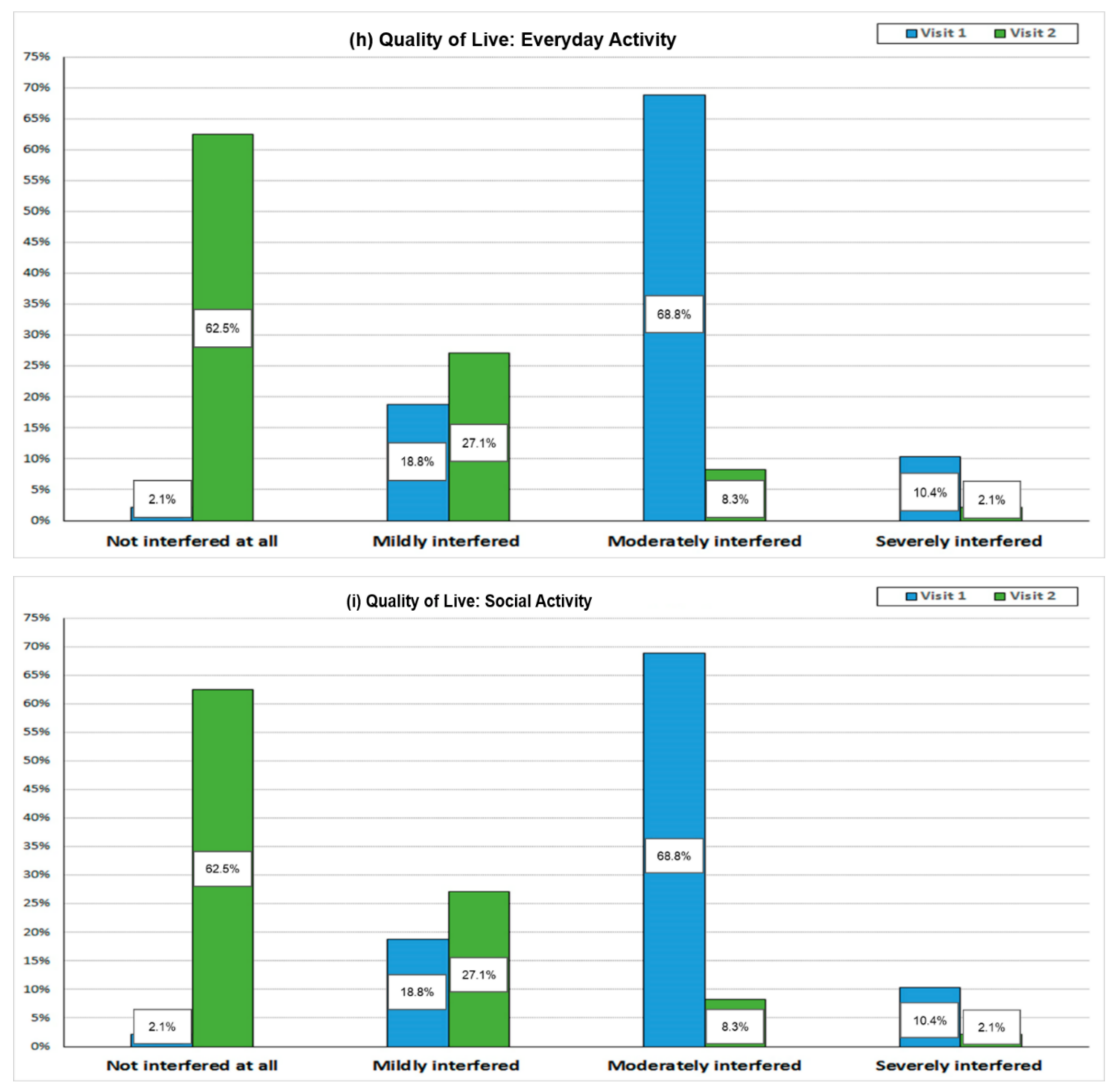
Distribution of scores at visit 1 and follow-up svisit in 48 female patients with AC. (**a**) Frequency; (**b**)Urgency; (**c**) Painful urination; (**d**) Incomplete bladder emptying; (**e**) Discomfort lower abdomen; (**f**) Blood in urine; (**g**) Quality of Life: General Discomfort; (**h**) Quality of Live: Everyday Activity; (**i**) Quality of Live: Social Activity.

In Table 3, the mean scores and 95% confidence intervals (CI), effect sizes correlation coefficient (r) of the ACSS items, and subscales in 48 female patients at first and follow-up visits are presented. The scores of all ACSS items and subscales were significantly (*p* < 0.05) reduced from the first visit to the follow-up visit, except for “urethral discharge” in the “Differential” domain. Figure 1a–i shows the distributions of scores in the Typical Symptoms and the Quality of Life domains in 48 female patients with AC at the first and the follow-up visit.

### 3.5. Correlation between Symptom Scores and Outcome

The correlations between the differences of various symptom scores and scores in ‘Dynamics’ found in 48 female patients between first and follow-up visit are shown in Table 4. The correlations were statistically significant (*p* < 0.05, two-sided) for all typical symptoms (except frequency), quality of life and the corresponding subscales. There were, however, no significant correlations between the differences of scores in the ‘Differential’ domain, such as flank pain, vaginal discharge, urethral discharge, and feeling febrile, which can be expected because these symptoms are only used for differential diagnosis and not for the patient-reported outcome.

### 3.6. Clinical Outcome Categories

The scores of the specific ACSS items at the follow-up visit and the correlations of the “Typical” and its subgroups, “Main Symptoms’”and “Five Typical Symptoms,” “Quality of Life,” “Typical” plus “Quality of Life,” and total ACSS domains with the scores of the “Dynamics” domain are shown in Table 5. The “Five Typical Symptoms” subscale of “Typical,” although not used for diagnostics, was also tested here for the outcome, because the sixth symptom (visible blood in urine) of “Typical” is only typical in the minority of patients suffering from a specific hemorrhagic cystitis. The results showed statistically significant correlations between the scores of all specific items and the corresponding subscales (except visible blood in urine) as mentioned before with the scores of the “Dynamics” domain.

### 3.7. Patient-Reported Outcome Assessment

**Figure 2:**
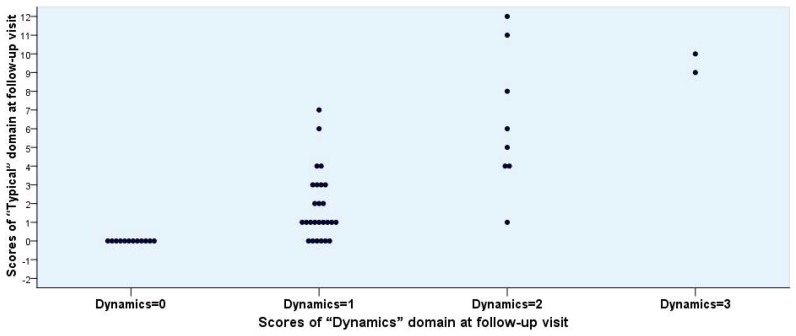
Individual scores for “Typical” versus “Dynamics” domain obtained from 48 female patients at the follow-up visit.

**Figure 3:**
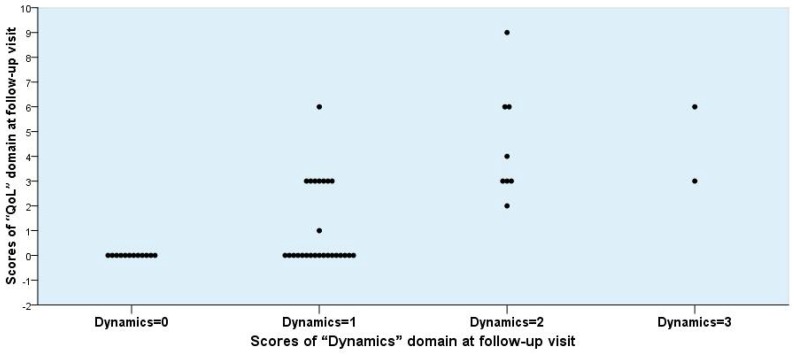
Individual scores for “Quality of Life (QoL)” versus “Dynamics” domain obtained from 48 female patients at the follow-up visit.

**Figure 4:**
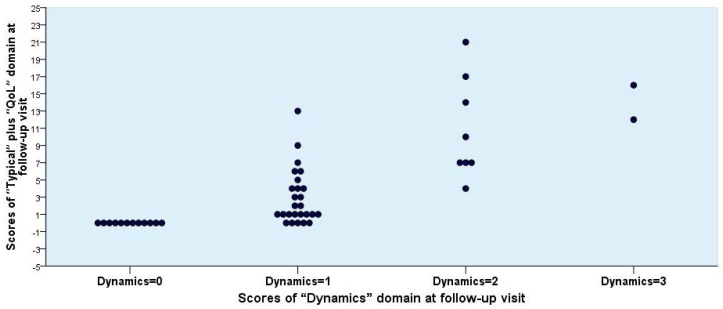
Individual scores for “Typical” plus “Quality of Life (QoL)” versus “Dynamics” domain obtained from 48 female patients at the follow-up visit.

To differentiate the patient-reported clinical outcome into the two categories, *Success* and *Non-success*, several rational possibilities may be discussed. The differentiation according to the scores obtained by the “Dynamics” domain alone was not convincing enough (Figure 2, Figure 3 and Figure 4). If *Success* would be defined only by a score of 0, the number would have been unrealistically too low. If, however, Success is defined as a score of 1 or less, then the number seems to be adequate (Table 6, Mode 1).

**Figure 5:**
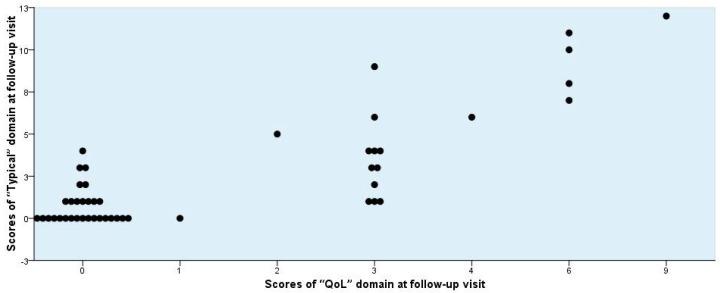
Individual scores for “Typical” versus “Quality of Life (QoL)” domain obtained from 48 female patients at the follow-up visit.

A differentiation between *Success* and *Non-success* can also be performed without using the “Dynamics” domain in various ways using the individual patients’ scores of the “Typical” and its subgroups, “Main Symptoms” and “Five Typical Symptoms,” “Quality of Life,” and “Typical” plus “Quality of Life” subscales, because a good correlation was seen between “Typical” and “Quality of Life” (Figure 5). In addition, it is reasonable to assume that in patients showing *Success* the scores of each specific item in the “Typical” or “Quality of Life” domains should not exceed 1 (mild) (Table 6).

In Table 6 the evaluation according to the four modes—Nr. 3, 4, 6, and 7—showed exactly the same numbers (37/11 *Success/Non-success*), which also represented the same patients. By the mode Nr. 2 (Main Symptoms) two additional patients were rated as a success, in total 39 patients. Both of these two patients were 30 years of age, had a total score in the “Typical” domain of 17, pyuria, bacteriuria of ≥10^5^ CFU/mL with *Escherichia coli* as uropathogen, and both also had visible blood in the urine with a score of 2 (moderate), thus having a hemorrhagic cystitis. At the follow-up visit at Day 3, one patient still had visible blood in her urine (severe) and suprapubic pain (moderate), although her total score of the Main Symptoms was reduced to 1 (mild painful urination). The other patient complaint at the follow-up visit at Day 5 about the moderate incomplete emptying of the bladder and suprapubic pain, although her total score of the Main Symptoms was reduced to 3 (mild frequency, urgency, and painful urination). Most likely in both cases, the follow-up visit was too early to demonstrate the final success of the treatment. Nevertheless, this evaluation demonstrates that with the Main Symptoms alone the success rate at a given visit may be overestimated at least in a few patients. We recommend to use the evaluation by the mode Nr. 4 (‘Typical’ domain score not more than 4 with no item more than 1)—or alternatively by the mode Nr. 3 (Five Typical Symptoms)—as standard to differentiate between *Success* and *Non-success,* which may be confirmed by either using the mode Nr. 6 or 7 including also the scores obtained in the “Quality of Life” domain.

## 4. Discussion

In the past the primary aim of clinical studies on female patients with uncomplicated acute cystitis (AC) was the eradication of bacteriuria at the test-of-cure visit and the clinical outcome was used as confirmation. In such a classical study [8] for inclusion a positive culture was defined as isolation of a uropathogen in quantities ≥10^5^ colony-forming units (CFU)/mL urine with pyuria, defined as ≥10 leukocytes/mm^3^, and bacteriologic response was assessed as *eradication* (<10^4^ CFU/mL of original uropathogen), *persistence* (≥10^4^ CFU/mL of original uropathogen), *superinfection* (≥10^5^ CFU/mL of a uropathogen other than the original pathogen at any time during active therapy), and *new infection* (≥10^5^ CFU/mL of a uropathogen other than the original pathogen at any time after the end of therapy).

In contrast, Stamm et al. [9] had already shown that the traditional diagnostic criterion, ≥10^5^ CFU/mL of midstream urine, has a very high degree of diagnostic specificity (99%) but a very low level of sensitivity (51%), which means that only 51% of symptomatic women with lower urinary tract infections (UTI) could be identified, whose bladder urine—obtained by suprapubic aspiration or by catheter—contained coliforms. The authors found the best diagnostic criterion to be ≥10^2^ CFU/mL (sensitivity, 95%; specificity, 85%) and suggested that clinicians and microbiologists should alter their approach to the diagnosis and treatment of women with acute symptomatic coliform infection of the lower urinary tract. In a more recent study, Hooton et al. [10] confirmed that colony counts of *E. coli* as low as even 10 to 10^2^ CFU/mL in midstream urine were sensitive and specific for the presence of *E. coli* in catheter urine in symptomatic women. Therefore, it is not surprising that in the study of Henry et al. [8] of the 469 patients not valid for efficacy, 90% (421) were excluded because no causative organism was isolated in predefined quantity (i.e., ≥10^5^ CFU/mL) before treatment. This number was about the same as the 422 patients evaluable for efficacy. This consideration shows that in the past only a highly selected group of patients with acute lower UTI were eligible for clinical studies, although most of the excluded patients may have had about the same symptomatology also caused by acute uncomplicated lower UTI.

Because the traditional diagnostic criterion of ≥10^5^ CFU/mL has a very low sensitivity, guidelines by the Infectious Diseases Society of America (IDSA) supported by the U.S. Food and Drug Administration (FDA) recommended to include also patients with a bacteriuria of ≥10^3^ CFU/mL of a uropathogen with only little loss of sensitivity (about 80%), but greater specificity (about 90%) as compared to the recommendations of Stamm et al. [9], because routine microbiological techniques can more reliably identify 10^3^ CFU/mL than 10^2^ CFU/mL [11]. On the other hand, to measure eradication of bacteriuria then became more difficult, because these microbiological techniques have at least an error probability of a decimal power. Therefore, many studies still used the much higher, but easier to handle threshold of 10^5^ CFU/mL as demonstrated above [8].

For the clinical inclusion in such a traditional study [8] each patient had to have ≥2 signs or symptoms suggestive of an acute uncomplicated UTI (i.e., dysuria, frequency, urgency, suprapubic pain) with an onset of symptoms within 72 h of enrollment. In the study mentioned, the urinary frequency was the most common symptom (97.6%), followed by urgency (95.0%), dysuria (89.6%), and suprapubic pain (89.6%). Most patients in this study reported that the intensity of their symptoms was mild to moderate, although urinary urgency was severe in 37.4% of patients.

Since in actual clinical practice, and also supported by recent guidelines [11,12,13], culture and susceptibility testing are not often performed in young to middle-aged women with acute uncomplicated UTI, the accurate diagnosis made only by the patient’s symptoms has become more important. Therefore the ACSS was developed and validated as a self-reporting questionnaire for diagnosing AC in female patients by assessing typical and differential symptoms, quality of life, and additional health conditions, which may play an important role in such a clinical setting [1,3]. As an optimal threshold to predict AC with 89.3% (95% CI; 81.0–93.7%) sensitivity and 92.5% (95% CI; 86.9–97.0%) specificity, respectively, a total score of 6 points in the domain of typical symptoms could be established in the reevaluated ACSS [3].

Classically clinical outcome is evaluated in such a study [8] on the signs and symptoms of UTI (see above) as *Cure* (disappearance of or improvement in signs and symptoms of the infection such that additional antimicrobial therapy was not required) and *Failure* (no apparent response to therapy, persistence of signs and symptoms of infection, reappearance of signs and symptoms at or before the test-of-cure visit, or use of additional antimicrobial therapy for the current infection). Since in the past clinical outcome was only supportive to the microbiological response, more exact clinical outcome measures were not necessary.

Since even therapeutic strategies of AC are today investigated in controlled randomized trials (RCTs) with only symptomatic versus antibiotic treatment, e.g., ibuprofen versus fosfomycin trometamol [14], reliable measures for the clinical outcome will become critical. The primary aim of such a study is now the patient-reported outcome and not the microbiological response. At least in a pilot study, it has been shown that persistent, but asymptomatic bacteriuria after successful symptomatic therapy does not necessarily trigger early recurrence [15]. In another study on young women with recurrent UTI it also could be established that treatment of asymptomatic bacteriuria between symptomatic episodes may even be harmful and trigger more frequent symptomatic recurrences and as expected is associated with a higher prevalence of antibiotic-resistant strains [16,17,18]. Therefore patient-reported outcome instruments need to be developed to differentiate more carefully between *Success/Cure* and *Non-success/Failure* to measure benefit or risk in medical product clinical trials as suggested by the FDA [19]. 

Defining *Success/Cure as* complete disappearance of all signs and symptoms caused by the infection would be ideal, but this cannot realistically be measured. Although the symptoms in the “Typical” domain are of course typical for AC, some patients will have similar symptoms at least of mild severity caused by other reasons. Although it can be assumed, that such kind of symptoms was already present before the onset of infection, reporting of subjective symptoms depends also on cultural behavior and present psychological conditions. Therefore, also in the past disappearance as well as improvement were used to define *Success/Cure*. The question remains how much improvement is necessary to define *Success*.

In the present study 48 female patients diagnosed with AC according to clinical and laboratory assessment and scoring by means of the ACSS (part A) about typical symptoms, differential symptoms, quality of life and additional conditions, were treated and the patient-reported outcome was evaluated with part B of the ACSS.

Considering the results in Table 6 the optimal evaluation would be to define *Success* only in patients with a score of 4 or less in the “Typical” domain (mode Nr. 4) with no specific score >1, which should be confirmed at the same time with a score of 3 or less in the “Quality of Life” domain with no specific score >1 (mode Nr. 7). Such a procedure includes a complete disappearance or at least such an improvement of the typical symptoms, that the interference on the specific items in the two domains is only mild at the same time. If there is an obvious discrepancy between the two domains (typical symptoms and quality of life), patients need to be fully assessed having in mind that (i) these symptoms are in fact typical for acute cystitis, but may also be found at least in part in patients with other diseases; and (ii) quality of life can also be altered not only by the symptoms of acute cystitis but also by underlying conditions of the patient.

The differentiation between *Success* and *Non-success* made only with the scores of the “Main Symptoms” showed that the success rate at a given visit may be overestimated at least in a few patients. Therefore, we recommended to define *Success* by a score of 4 or less in the “Typical” domain or in the “Five Typical Symptoms” domain (mode 3) with no item more than 1 as standard, which then could be also confirmed by the scores obtained in the “Quality of Life” domain as outlined in Table 6. The reason why the same scoring threshold can be used for mode 3 and 4 is explained by the fact, that all patients finally rated as Success had a score of 0 in the subscale “visible blood”. This finding may also have clinical significance in that way that a patient at follow-up (e.g., test of cure) with total scores suggesting Success but with a score of ≥1 in the subscale “visible blood” should be investigated more thoroughly whether the hematuria might have been caused by other underlying urological or nephrological diseases than by hemorrhagic cystitis.

According to this recommended evaluation, 37 (77.1%) patients would be rated as *Success* and 11 (22.9%) patients as *Non-success* in the present study. At the first glance, such a *Success* rate seems to be lower than reported in most of the clinical trials, but one has to consider that these patients were not treated uniformly according to the most effective strategy and a fixed follow-up visit was not scheduled as part of the study following practice guidelines. Therefore, it can be assumed that many patients treated successfully did not return for a follow-up visit because the purpose of this study was to evaluate the practicability of the ACSS in everyday practice.

The study has also shown that simple overall summary questions like in the “Dynamics” domain and used in many past clinical studies, but never successfully validated, are not sufficient to differentiate between *Success* and *Non-success.* There was, however, a significant correlation with the score reduction in the specific items of the “Typical” and “Quality of Life” domains and the corresponding subscales. Several potential reasons for the lack of clear differentiation between the *Success* and *Non-success* can be discussed. In the present study, the follow-up visit was on average scheduled earlier than the “test-of-cure” visit is usually scheduled, so relapsed patients may not be accounted for and more patients with resolution of symptoms of AC are included in the sample. There were only 10 out of 48 patients with higher values of the scores obtained from the “Dynamics” domain (scores equal to 2 or 3, none for 4), thus, small numbers and an uneven split may have led to the lack of differentiation between *Success* and *Non-Success*. A further reason might be, that the questions in the “Dynamics” domain were not precise enough and need to be improved. To even increase the applicability of the ACSS slight modifications of the questions are suggested as follows:

Score 0: Now I feel back to normal (*All symptoms are gone*);
Score 1: Now I feel much better (*Most of the symptoms are gone*);
Score 2: Now I feel only somewhat better (*Only some of the symptoms are gone*);
Score 3: Now, there are barely any changes (*I have still about the same symptoms*);
Score 4: Now, I feel worse (My condition is worse)

Nevertheless, the present ACSS appeared to be a suitable instrument for patient-reported outcome measures because a clear and well-balanced differentiation between *Success* and *Non-success* can be performed. A further advantage of the ACSS would be to use it as patient’s diary to measure the time of the symptoms declining or how many patients reach a certain predefined goal. Such an instrument may be especially useful in controlled RCTs to assess not only differences in clinical efficacy at certain predefined visits but to get results almost every day to determine the effectiveness of different therapeutic strategies.

## 5. Conclusions

The ACSS has proven to be not only a useful instrument to clinically diagnose AC in women by assessing the severity of symptoms and their impact on quality of life as well as to differentiate AC from other urogenital disorders. It is also a suitable instrument for patient-reported outcome measures, with applicability both in daily practice and clinical studies. The results of the study indicate that modifications in the “Dynamics”domain may even increase the applicability.

## 6. Patents: Copyright and Translations of the ACSS in Other Languages

The ACSS is copyrighted by the Certificate of Deposit of Intellectual Property in Fundamental Library of Academy of Sciences of the Republic of Uzbekistan, Tashkent (Registration number 2463; 26 August 2015) and the Certificate of the International Online Copyright Office, European Depository, Berlin, Germany (Nr. EU-01-000764; 21 October 2015). The Rightholders are Jakhongir Fatikhovich Alidjanov (Uzbekistan), Ozoda Takhirovna Alidjanova (Uzbekistan), Adrian Martin Erich Pilatz (Germany), Kurt Günther Naber (Germany), Florian Martin Erich Wagenlehner (Germany). http://www.avtor-depository.com/index.php?option=com_desposition&task=display_desp_det&id=2612&lang=ru (assessed on 21 May 2018); http://interoco.com/all-materials/work-of-science/1013-1951954939.html (assessed on 19 February 2018). The e-USQOLAT is copyrighted by the Authorship Certificate of the International Online Copyright Office, European Depository, Berlin, Germany (Nr. EC-01-001179; 18 May 2017). http://inter.interoco.com/copyright-depository/computer-programs/1438-2017-05-18-10-59-16.html?path=computer-programs (assessed on 10 February 2018). Translations of the ACSS in other languages are available on the website: http://www.acss.world/downloads.html. 

## Figures and Tables

**Table 1:** Answers from 48 female patients at follow-up visit in the “Dynamics” domain of part B of the Acute Cystitis Symptom Score (ACSS) Questionnaire.

Score	Answer	Follow-Up Visit *n* = 48 (100%)
0	Yes, I feel myself great better (All symptoms went off)	12 (25.0%)
1	Yes, I feel myself much better (Majority of symptoms were solved)	26 (54.2%)
2	Yes, I feel myself somewhat better (Some symptoms are remaining)	8 (16.7%)
3	No changes, I feel about the same (All symptoms are remaining)	2 (4.2%)
4	Yes, I feel worse (My condition is declining)	0 (0.0%)

**Table 2:** Values of current internal consistency, alpha ‘if item deleted’ and item-total correlations for ACSS items.

Items of the ACSS	Correlation Between Item and Entire ACSS		
Dynamics		Dynamics	Cronbach’s Alpha If Item Deleted
Typical	Frequency	0.83	0.91
Typical Differential	Urgency	0.62	0.92
	Painful urination	0.63	0.91
	Incomplete bladder emptying	0.77	0.91
	Suprapubic pain	0.63	0.91
	Visible blood in urine	0.80	0.91
	Flank pain	0.40	0.92
Differential Quality of Life (QoL)	Vaginal discharge	0.52	0.93
	Urethral discharge	0.19	0.93
	Feeling of chill/fever	0.40	0.92
	General discomfort	0.40	0.92
QoL	Impairment of everyday activity	0.82	0.91
	Impairment of social activity	0.86	0.91

Current Cronbach’s alpha = 0.92 (95% CI: 0.89 to 0.95).

**Table 3:** Mean scores and 95% confidence intervals (CI), effect sizes correlation coefficient (r) of ACSS items, and subscales in 48 female patients at first and follow-up visit.

**ACSS Items**		**Mean Scores (95% CI)**			***p*-Value**	**Effect Size *r***
		**First Visit**	**Follow-Up Visit**	**Difference Between Scores**		
Typical	Frequency	2.06 (1.82 to 2.31)	0.52 (0.29 to 0.75)	−1.68 (−1.94 to −1.42)	0.000	−0.82
	Urgency	2.02 (1.75 to 2.29)	0.46 (0.23 to 0.69)	−1.56 (−1.87 to −1.26)	0.000	−0.79
	Painful urination	2.29 (2.08 to 2.51)	0.46 (0.28 to 0.64)	−1.83 (−2.10 to −1.57)	0.000	−0.85
	Incomplete bladder emptying	1.92 (1.70 to 2.13)	0.44 (0.21 to 0.66)	−1.48 (−1.74 to −1.22)	0.000	−0.82
	Suprapubic pain	1.81 (1.58 to 2.05)	0.44 (0.25 to 0.63)	−1.38 (−1.63 to −1.12)	0.000	−0.82
	Visible blood in urine	0.71 (0.45 to 0.96)	0.15 (−0.02 to 0.32)	−0.56 (−0.84 to −0.29)	0.001	−0.50
Differential	Flank pain	1.27 (0.99 to 1.55)	0.60 (0.41 to 0.80)	−0.67 (−0.92 to −0.42)	0.000	−0.61
	Vaginal discharge	0.38 (0.18 to 0.57)	0.19 (0.03 to 0.34)	−0.19 (−0.33 to −0.05)	0.013	−0.36
	Urethral discharge	0.25 (0.10 to 0.40)	0.08 (0.00 to 0.16)	−0.17 (−0.34 to 0.01)	0.059	−0.27
	Feeling of chill/fever ^a^	0.26 (0.09 to 0.43)	0.07 (−0.01 to 0.14)	−0.20 (−0.34 to −0.05)	0.014	−0.36
QoL	General discomfort	2.04 (1.86 to 2.22)	0.52 (0.30 to 0.74)	−1.52 (−1.78 to −1.26)	0.000	−0.82
	Impairment of everyday activity	1.88 (1.70 to 2.05)	0.50 (0.28 to 0.72)	−1.38 (−1.63 to −1.12)	0.000	−0.81
	Impairment of social activity	1.77 (1.57 to 1.97)	0.50 (0.28 to 0.72)	−1.27 (−1.52 to −1.03)	0.000	−0.80
**ACSS Subscales**		**Mean total Scores and (95% CI)**			***p*-Value**	**Effect Size *r***
		**First Visit**	**Follow-Up Visit**	**Difference Between Scores**		
“Main Symptoms” ^b^		6.37 (5.85 to 6.90)	1.44 (0.90 to 1.98)	−4.94 (−5.56 to −4.31)	0.00	−0.86
“Five Typical Symptoms” ^c^		10.10 (9.27 to 10.93)	2.31 (1.44 to 3.18)	−8.75 (−8.75 to −6.84)	0.00	0.86
“Typical”		10.81 (9.89 to 11.73)	2.46 (1.52 to 3.39)	−8.35 (−9.42 to 7.29)	0.00	−0.86
“Differential” ^a^		2.13 (1.66 to 2.61)	0.85 (0.51 to 1.19)	−1.28 (−1.63 to −0.94)	0.00	−0.77
“Quality of Life (QoL)”		5.69 (5.21 to 6.17)	1.52 (0.87 to 2.17)	−4.17 (−4.86 to −3.47)	0.00	−0.82
“Typical” and “QoL”		16.50 (15.24 to 17.76)	3.98 (2.45 to 5.51)	−12.52 (−14.15 to −10.89)	0.00	−0.86
Total ACSS ^a^		18.37 (16.97 to 19.77)	4.65 (2.93 to 6.37)	−13.72 (−15.46 to −11.98)	0.00	−0.86

^a^ Based on sum of scores of 46 cases with non-missing values; ^b^ “Main Symptoms” include “Typical” 1–3: frequency, urgency, painful urination; ^c^ “Five Typical Symptoms” includes “Typical” with the exclusion of one symptom (visible blood in the urine).

**Table 4:** Correlation between scores of “Dynamics” domain and differences in scores for ACSS items in 48 female patients between first and follow-up visit.

**ACSS Items**		**Spearman’s Rho**	***p*-Value ^a^**
Typical	Frequency	0.27	0.067
	Urgency	0.34	0.017
	Painful urination	0.4	0.005
	Incomplete bladder emptying	0.375	0.009
	Suprapubic pain	0.409	0.004
	Visible blood in urine	0.301	0.037
Differential ^b^	Flank pain	0.079	0.594
	Vaginal discharge	0.061	0.682
	Urethral discharge	−0.059	0.689
	Feeling of chill/fever	0.088	0.563
QoL	General dyscomfort	0.581	0.000
	Impairment of everyday activity	0.613	0.000
	Impairment of social activity	0.499	0.000
**Differences in scores for ACSS Subscales**		**Spearman’s Rho**	***p***
“Main Symptoms” ^c^		0.435	0.002
“Five Typical Symptoms” ^d^		0.514	0.000
“Typical”		0.508	0.000
“Differential”		0.152	0.312
“QoL”		0.606	0.000
“Typical” plus “QoL”		0.598	0.000
Total ACSS score		0.624	0.000

QoL-Quality of Life; ^a^ significant = *p* ≤ 0.05; ^b^ Non-obligatory item “Hyperthermia” (Please indicate if measured) was not included into the analysis because majority of patients (60–95.2%) missed to check this item during their follow-up visit; ^c^ “Main Symptoms” include “Typical” 1–3; frequency, urgency, painful urination; ^d^ “Five Typical Symptoms” include “Typical” with the exclusion of one symptom (visible blood in the urine).

**Table 5:** Mean scores and 95% confidence intervals (CI) of ACSS items and subscales in 48 female patients according to scores of the “Dynamics” domain at the follow up visit.

ACSS Items		Mean Scores (95% CI)					*p*-Value
		Total	Dynamics = 0	Dynamics = 1	Dynamics = 2	Dynamics = 3	Dynamics
		*n* = 48	*n* = 12	*n* = 26	*n* = 8	*n* = 2	0 + 1 vs. 2 + 3
Typical	Frequency	0.52 (0.29 to 0.75)	0.00 (constant)	0.46 (0.20 to 0.72)	1.38 (0.49 to 2.26)	1.00 (−11.71 to 13.71)	0.008
	Urgency	0.46 (0.23 to 0.69)	0.00 (constant)	0.35 (0.15 to 0.54)	1.13 (−0.01 to 2.26)	2.00 (constant)	0.018
	Painful urination	0.46 (0.28 to 0.64)	0.00 (constant)	0.35 (0.15 to 0.54)	1.13 (0.83 to 1.42)	2.00 (constant)	0.000
	Incomplete bladder emptying	0.44 (0.21 to 0.66)	0.00 (constant)	0.35 (0.12 to 0.57)	1.00 (0.00 to 2.00)	2.00 (constant)	0.018
	Suprapubic pain	0.44 (0.25 to 0.63)	0.00 (constant)	0.35 (0.12 to 0.57)	1.13 (0.59 to 1.66)	1.50 (−4.85 to 7.85)	0.000
	Visible blood in urine	0.15 (−0.02 to 0.32)	0.00 (constant)	0.00 (constant)	0.63 (−0.37 to 1.62)	1.00 (−11.71 to 13.71)	0.154
QoL	General dyscomfort	0.52 (0.30 to 0.74)	0.00 (constant)	0.38 (0.15 to 0.62)	1.50 (0.73 to 2.27)	1.50 (−4.85 to 7.85)	0.000
	Impairment of everyday activity	0.50 (0.28 to 0.72)	0.00 (constant)	0.35 (0.12 to 0.57)	1.50 (0.87 to 2.13)	1.50 (−4.85 to 7.85)	0.000
	Impairment of social activity	0.50 (0.28 to 0.72)	0.00 (constant)	0.35 (0.12 to 0.57)	1.50 (0.87 to 2.13)	1.50 (−4.85 to 7.85)	0.000
**ACSS subscales**		**Mean scores (95% CI)**					
“Main Symptoms” ^a^		1.44 (0.90 to 1.98)	0.00 (constant)	1.15 (0.72 to 1.59)	3.63 (1.63 to 5.62)	5.00 (−7.71 to 17.71)	0.000
“Five Typical Symptoms” ^b^		2.31 (1.44 to 3.18)	0.00 (constant)	1.85 (1.10 to 2.59)	5.75 (2.73 to 8.77)	8.50 (−10.56 to 27.56)	0.000
“Typical”		2.46 (1.52 to 3.39)	0.00 (constant)	1.85 (1.10 to 2.59)	6.38 (3.25 to 9.50)	9.50 (3.15 to 15.85)	0.000
“Quality of Life (QoL)”		0.85 (0.51 to 1.19)	0.00 (constant)	1.08 (0.40 to 1.75)	4.50 (2.55 to 6.45)	4.50 (−14.56 to 23.56)	0.000
“Typical” and “QoL”		1.52 (0.87 to 2.17)	0.00 (constant)	2.92 (1.63 to 4.22)	10.88 (5.95 to 15.80)	14.00 (−11.41 to 39.41)	0.000
Total ACSS		3.98 (2.45 to 5.51)	0.42 (−0.01 to 0.84)	3.08 (1.97 to 4.20)	12.63 (7.51 to 17.74)	17 (constant)	0.000

^a^ “Main Symptoms” include “Typical” 1–3; frequency, urgency, painful urination; ^b^ “Five Typical Symptoms” include “Typical” with the exclusion of one symptom (visible blood in the urine).

**Table 6:** Various possibilities to differentiate between *Success* and *Non-success* in 48 female patients treated for acute uncomplicated cystitis using part B of the ACSS. QoL = Quality of Life; N—number.

Mode	Domain (s)	Definition of *Success*	*Success*	*Non-Success*
		(Scores)	N (%)	N (%)
1	Dynamics	≤1	38 (79.2%)	10 (20.8%)
2	Main Symptoms ^a^	≤3, but no item >1 (mild)	39 (81.2%)	9 (18.8%)
3	Five Typical Symptoms ^b^	≤4, but no item >1 (mild)	37 (77.1%)	11 (22.9%)
4	Typical	≤4, but no item >1 (mild)	37 (77.1%)	11 (22.9%)
5	QoL	≤3, but no item >1 (mild)	42 (87.5%)	6 (12.5%)
6	Typical plus QoL	≤7, but no item >1 (mild)	37 (77.1%)	11 (22.9%)
7 ^c^	Typical/QoL	≤4/≤3, but no item >1 (mild)	37 (77.1%)	11 (22.9%)

^a^ “Main Symptoms” include “Typical” 1–3: frequency, urgency, painful urination; ^b^ “Five Typical Symptoms” include “Typical” with the exclusion of one symptom (visible blood in the urine); ^c^ recommended as overall optimal balanced to differentiate between *Success* and *Non-Success*.

## Supplementary Materials

Figure S1: Updated Uzbek (Cyrillic) version of the Acute Cystitis Symptom Score (ACSS) for the follow-up visit (part B). Website: http://www.acss.world/ACSS_UZ_C.pdf (assessed on 10 February 2018), Figure S2: Updated Russian version of the Acute Cystitis Symptom Score (ACSS) for the follow-up visit (part B). Website: http://www.acss.world/ACSS_RU.pdf (assessed on 10 February 2018).
